# Introspection of the Etiopathological Mechanisms Underlying Noncarious Cervical Lesions: Analysis of the Different Theories and Their Impact on the Mineralized Structures of the Tooth

**DOI:** 10.1155/2023/8838314

**Published:** 2023-11-06

**Authors:** Mario Dioguardi, Davide Polverari, Francesca Spirito, Giovanna Iacovelli, Diego Sovereto, Enrica Laneve, Giorgia Apollonia Caloro, Andrea Ballini, Lorenzo Lo Muzio

**Affiliations:** ^1^Department of Clinical and Experimental Medicine, University of Foggia, Via Rovelli 50, 71122, Foggia, Italy; ^2^Unità Operativa Nefrologia e Dialisi, Presidio Ospedaliero Scorrano, ASL (Azienda Sanitaria Locale) Lecce, Via Giuseppina Delli Ponti, 73020, Scorrano, Italy

## Abstract

The noncarious cervical lesions (NCCLs) recognize an etiological framework of onset very different from the carious processes with etiology whose bacteria aggregated in a biofilm play a predominant role, leading in this way to the loss of the mineralized structure of the tooth. The pathological picture of the NCCLs, which manifests itself with a clinical picture of dental wear, differs from caries because it mainly recognizes a series of pathological processes, such as erosion, through the action of generally acidic chemical agents and abrasion, which is basically expressed through repeated mechanical trauma characteristic of tooth brushing. However, in the literature, there is no unanimous agreement in identifying only these two mechanisms, but there are some who propose a more marked role of anomalous occlusal loads, which would be unloaded on some teeth which, in addition to both erosive and abrasive mechanisms, would give rise to abfraction. Therefore, the aim of this review was to collect literature etio-pathological information and discuss the mechanisms underlying NCCLs.

## 1. Introduction

Caries is one of the most common pathologies in the world, affecting about 90% of the adult population. Caries recognizes a multifactorial etiology and manifests itself as a process of destruction of the tooth's hard tissues, such as enamel and dentin, by acidic byproducts derived from dietary carbohydrates bacterial fermentation [1].

The factors determining the appearance of carious pathology are, first, bacterial factors and factors related to diet; in fact, the quantity and frequency of carbohydrates introduced with diet are directly proportional to the risk of developing a carious lesion; therefore, acidogenic (acid-producing) oral plaque bacteria, ferment carbohydrates that are introduced into the mouth, producing organic acids, including lactic, formic, acetic, and propionic [2]. In addition, salivary factors, specifically the quantity and quality of saliva produced, were involved in this process. Poor saliva production and an acid pH are important predisposing factors since the physiological function of cleaning dental surfaces is lost. Finally, other factors, such as drug intake, previous experience of carious lesions, addiction to smoking, alcohol, and drugs, are involved in the process [3].

In general, the mechanisms of formation and evolution of dental caries can be described as follows: the bacterial plaque present, in particular Streptococci of the Mutans group as well as Lactobacilli, were responsible for the carbohydrates fermentation process and, as a consequence, there is a production of organic acids such as lactic acid and/or formic acid [2].

Besides, through diffusion processes in the enamel, dentin, and cementum, are dissolve the mineral crystals, causing the demineralization phenomena, with consequent dental surface abrading, and the formation of a cavity.

The initial stages of enamel caries involve the formation of a white spot lesion [2, 4].

However, the described etiopathogenetic aspects cannot justify the onset of all the lesions affecting the teeth's hard structures, such as abrasions and erosions, which can be identified as causes related to chemical aggression (acid substances) or mechanical trauma such as brushing or para-functions such as bruxism. The theme of the etiological classification, with an appropriate classification of the lesions, has been addressed several times over the years.

In 1908, the US dentist Greene Vardiman Black [5] proposed to the international medical community the first coding and classification of carious lesions, which was then universally accepted.

The classification proposed by Black divides the coronal carious lesions into five classes based on the location, the degree of involvement of the tooth tissue, the compromised tooth, and the evolution of the lesion [5].

However, this classification did not take into consideration the etiopathogenesis of all dental coronal lesions but mainly considered the residual cavity remaining once the pathological tissue affected by the carious process has been eliminated. In fact, the noncarious cervical lesions (NCCLs) are a typical dental lesion affecting the coronal cervical region of the tooth which is included in the V classes of Black but which does not recognize a multifactorial etiology described for the carious process or the traumatic event.

Black identified these lesions as erosions, distinguishing them from abrasions affecting the occlusal surface of the teeth; the shapes of the lesions, such as dish-shaped areas, wedge-shaped areas [6], flattened areas, irregular areas, and figured areas, included a series of etiological causes, i.e., friction from brushing trauma, enamel development defects, that make prisms more susceptible to erosion and acid aggression of the enamel.

The most recent systematic reviews have mainly highlighted the clinical and therapeutic aspects, emphasizing, which were the adhesive and restaurative strategies that guaranteed the greatest stability and time duration [7–14]; on the other hand, further reviews have focused on hypersensitivity [15] and the prevalence of these lesions [14]. Only two reviews have systematically highlighted some etiopathogenetic aspects of NCCLs, including occlusal stresses [16, 17].

The purpose of this literature review was to describe the etiopathogenesis of NCCLs through the analysis of the medical literature, highlighting the several controversies that characterize the onset of these lesions.

## 2. Materials and Methods

The drafting of this narrative review followed the indications of the SANRA (Scale for the Assessment of Narrative Review Articles) [18].

For the drafting of this review, the sources PubMed, Scopus, ScienceDirect, and Google Scholar were considered, and the search was conducted with keywords such as “Non-Carious Cervical Lesions,” “Abfraction,” “cervical wedge-shaped lesion,” and “tooth erosion.” The research was conducted between June 1, 2023 and June 8, 2023 and was conducted by M.D., and all the studies written in English investigating the etiopathogenesis of NCCLs were taken into consideration by applying language filters.

## 3. Discussion

### 3.1. Terminology

Starting from the terminology of the pathological processes, there is still today a nonunivocal use of some terms aimed at identifying both the clinical conditions and the pathological processes implemented in the formation of the NCCLs.

From a meeting between the European Organization for Research on Dental Caries and the Cariology Research Group of the International Association for Dental Research with the aim of defining the terminology of erosive tooth wear and dental caries, some terms emerged that were largely accepted anonymously by all participants in defining the several situations [19].

The terminology used that has found the greatest consensus for clinical conditions is the most generic “Tooth wear and Erosive tooth wear.” The first is defined as “the cumulative surface loss of mineralized tooth substance due to physical or chemo-physical processes (dental erosion, attrition, abrasion),” and all clinical situations caused by trauma, caries, and resorptions are excluded. On the other hand, erosive tooth wear defines all the clinical situations in which the loss of mineralized hard tissue is due to dental erosion phenomena, which is defined as the chemical loss of mineralized tooth substance caused by exposure to acids not derived from oral bacteria. If the loss is due to a physical force implemented by contact with the opposing tooth, it is so-called friction, while if the cause is an object other than a tooth, the process is called dental abrasion.

Abfraction is the pathological loss of tooth substance caused by biomechanical loading forces that cause enamel and dentin to bend and fail at a location away from the load.

This term was introduced to explain the wear of the cervical tract of the tooth induced by the occlusal load; indeed, it is thought that it is caused by mechanical stresses that are extrinsic during chewing or in the course of malocclusion.

The latest indications also report not to recommend the term abfraction because the level of evidence is lacking in data to be able to justify a pathological process separate from the conditions previously described [19].

### 3.2. Dental Erosions

Dental erosions are a process that leads to the loss of only the superficial dental hard structure through chemical dissolution and not for a bacterial cause of proteolytic enzymes concentrated in a biofilm adhered to the dental surface [20].

The chemical degradation macroscopically manifests itself first of all on the dental element, presenting itself with a different severity based on the strength of the erosive attacks (pH, concentration of calcium and phosphate ions in saliva, its chelating properties), and occurs on the hydroxyapatite crystals [21].

There are several indices that evaluate the changes in the appearance of the dental hard tissues based on the severity of the strength of the erosive attacks in order to make their identification more immediate [22].

The most used classification for the physical examination involves the subdivision into the vestibular, oral, and occlusal surfaces, assigning a value of 4° to the vestibular surfaces (from 0 to 3, Table 1) and to the oral and occlusal surfaces of 3° (from 0 to 2, Table 2) [23].

Most of the erosive lesions are determined by the association of various promoting factors, which also depend on the various individual characteristics, including the salivary flow and its buffering power [24].

It is essential to identify these phenomena in order to carry out a correct differential diagnosis (with other types of cervical lesions) and for a correct therapeutic plan.

The different presence of intrinsic and extrinsic causes will be fundamental [23].

The most frequent patient-dependent causes are recurrent episodes of vomiting in patients undergoing therapy with cytostatic drugs or conditions of anorexia or bulimia [25].

In these situations, the appearance of erosions will manifest itself in a characteristic way initially at the level of the palatal surfaces of the dental elements of the upper arch and, subsequently, in more advanced stages, at the level of the occlusal surfaces of the lateral-posterior dental elements and finally on the buccal surfaces of the upper incisors [26].

Another frequent cause of erosion is represented by the conditions of gastroesophageal reflux, which, due to an insufficiency of the lower esophageal sphincter, determines repeated contact of the dental surfaces with the gastric acids that reach the oral cavity pushed by the abdominal positive pressure [27, 28].

In addition, there are numerous cases of erosion in patients who undergo radiotherapy in the head and neck area, with consequent insufficiencies of the salivary glands [29] (especially major ones, as in Sjogren's syndrome), also taking drugs that inhibit salivary secretion (i.e., antihistamines, antiemetics, etc.) [30].

The extrinsic causes concern eating habits (frequent and abundant intake of acidic foods or drinks such as fruit juices and/or even fruits rich in citric acid) [31]; the use of acid-based oral hygiene products, the use of acidic medicines, such as effervescent vitamin C or aspirin [32–34].

The erosive property of an acidic drink is determined by its low pH, chelating properties, duration, and frequency of use [35].

Some studies present in the literature explain that the main cause of dental abrasion [36–40], traumatic brushing, carries out its action on hard tissues softened by previous erosion phenomena, allowing the mechanical trauma to produce lesions more quickly [41]. In the case of alcoholic patients, the percentage tends to grow in recent years, and the incidence has increased, especially in men aged between 20 and 30 years [42].

Situations of this type present a very high risk of erosion since frequent vomiting is added to the immoderate intake of acidic alcoholic beverages [43].

Inhalation of acid gases or immersion in incorrectly chlorinated swimming pools can also induce erosion due to the formation of hydrochloric acid [44].

The erosive process begins at the level of the enamel tissue, and this phenomenon follows successive alternating phases [45], where enamel prisms are affected first, and interprismatic areas are affected second [46].

The patient is still asymptomatic in this phase, although not yet showing signs of erosion, exposing the site to the risk of mechanical wear, with a consequent increase in the adhesiveness of the bacteria.

Some in vitro studies conducted on human and bovine enamel demonstrate that when the dentin tissue is exposed, the histopathological scenario presents several new variables due to the reactivity of the anatomical site [47]. Based on the study model of the dentinal tubules (closed or open) and the hypersensitivity reported by the patient (absent or present), we distinguish two main types of dentin tissue (insensitive sclerotic and sensitive dentin) [48].

In this way, we will distinguish the early phase of the erosive process from the advanced one, establishing the most suitable conservative therapy.

The first studies were conducted in 1989 by the group led by Yoshiyama through the use of the electron microscope, performing biopsies of dentin lesions [49] and evaluating the characteristics of what we can now define as sensitive dentin [50].

These studies led to examining the dentin surface with many open tubules and with intact odontoblastic processes and unchanged anatomy; almost all erosions are symptomatic with a different perception by the patient ranging from a slight sensation of discomfort to long episodes of pain [51].

The etiology of hypersensitivity is explained with the hydrodynamic theory proposed by Brannstrom [52], who described that the development of the chemical, physical, thermal, or bacterial stimulus determines the movement of the dentinal fluids above the physiological limits, stimulating the mechanoreceptors associated with the A*δ* fibers present inside the pulp [53].

The fibers can, therefore, be stimulated by the lengthening of the odontoblastic process. The activation of the painful process occurs when the opening of a closed system occurs, such as the tubulo-dentinal system, causing the activation of the nerve fibers [54].

Following the conservative treatment, the patient reports improved symptoms, thus confirming the existence of the hydro-dynamic theory [50].

At the level of the dentin, modifications were appreciated both on the pulpal side and in the intra- and peritubular area, and factors, such as age and pathogenic stimuli, determine pulp maturation and responsiveness [55].

By definition, secondary dentin is the tissue that [56] is placed on the deepest surface of the tooth, causing a continuous reduction in the size of the pulp chamber, a phenomenon that is very evident in elderly patients [57].

The aforementioned tissue appears less organized than that defined as primary dentin developed during odontogenesis.

Tertiary dentin is defined as tissue with a very irregular appearance and often without dentinal tubules, which is deposited inside the pulp chamber exclusively in the areas subjected to irritative stimuli such as caries, conservative dental treatments, and friction phenomena.

The spaces between the tubules can be occluded due to the application of dentin or as a result of the precipitation of salts, which leads to a reduction of hypersensitivity [58, 59].

The different chromatic aspects, visible in the objective examination, are determined by the presence of calcified deposits present inside the tubules, which give them the same percentage of refraction typical of the intertubular dentin, making the dentin, especially in older subjects, dark yellow-brown color. This process affects the entire thickness of the dentin [60].

The study by Meurman and Frank concerned the progression of erosions present throughout the thickness of the dentin [61] and demonstrated that the first zones affected by the erosion process are the peritubular dentin zones [62].

They also demonstrated that if acid exposure is prolonged [63], the intertubular areas will be eroded, producing an increase in the diameter of the dentinal tubules, resulting in the increased sensitivity to external stimuli [64]; moreover, in dentin, a demineralized layer of ∼20 *µ*m thick can be formed during erosion, and with continuous exposure to acid this layer becomes thicker [65–67].

Besides, the two main protective factors of the development of enamel erosions should be analyzed: saliva and the chemical composition of the enamel.

The secretion of saliva stimulated by the presence of acids performs the function of diluting the concentration of the same, favoring by inducing swallowing, their more rapid elimination [68].

Besides, in addition to saliva, there are other buffer systems that inhibit acid, calcium, and phosphate levels [69]; these factors can potentially slow down the erosion process, both by being able to be used as substrates instead of the tooth surface and by determining partial remineralization.

Even the mucins, through a process of formation of the acquired film, inhibit the acid action by acting as a protection against the tooth surfaces against the dissolution processes [70].

The solubilization processes of the enamel vary according to the calcium concentration, and for this reason, they are called calcium-dependent. The main reacting groups of hydroxyapatites (phosphates and hydroxyl groups) contain, in fact, protons that react with the two unpaired electrons of the calcium ions [70].

In conditions of neutral pH, a balance is created inside the saliva between calcium and phosphate ions, a factor which allows the apatite not to undergo modifications from the state of oversaturation [71]; but if the salivary pH occurred to drop to critical levels for the enamel (pH 5.5), the dissolution phenomena begin [72].

The pH of the enamel can be brought to values close to 4.5 by the fluorapatite, causing the dissolution phenomena to be delayed.

### 3.3. Dental Abrasion

Dental abrasion appears as a wedge-shaped defect, with a smooth, hard, and shiny background, at the level of the cervical portion of the tooth [39], and the main cause is represented by the execution of an incorrect brushing technique, which can also determine the appearance of gingival recession [73].

It is necessary to be able to distinguish and define abrasion from erosion, as differentiation is fundamental for the choice of treatment.

While erosions are produced by the action of acids and abrasions can become a contributing cause, in the case of proper abrasion, the lesion is produced exclusively by trauma, and the softening with acid substances is superimposed only later.

This distinction becomes fundamental both from an etiological point of view and in the planning of primary and secondary prevention [74].

Often, not even an accurate anamnesis can guide us on the succession of lesions such as erosion, friction, and abrasion, and the patients themselves are unable to establish the chronological occurrence of the same, also because the micromorphology of abrasions has its own different characteristics compared to erosions and friction, causing there to be differences in the prognosis and progression [75].

The microscopic features of these similar lesions are hypermineralization of the intertubular dentin, degeneration of the peritubular dentin, precipitation of inorganic substance within the tubules, and the odontoblastic processes [76].

In certain situations, the terminal part of the Tomes fibers produces an organic dentin matrix, which then mineralizes, in response to abrasion, depositing itself in the tubular lumen, in a centripetal way, until it is completely closed [77].

This type of pulp defense, which does not differ much from what occurs in carious processes, is responsible for a different type of adhesion [78]. This category of reaction does not involve the intertubular dentin; therefore, in the case of esthetic restoration, this tissue is eliminated with the bur, leaving a smooth surface covered by what is defined as acquired cuticle [79] (salivary residues and other substances derived from the oral) [80].

It has been demonstrated that, in order not to alter the adhesion capacity of the restoration, the acquired cuticle must necessarily be removed [81, 82].

### 3.4. The Theory of Abfraction

“Abfraction” is a term not yet accepted by the scientific community, although it is widely used by many to indicate characteristic wedge-shaped lesions that generally affect the premolars buccal surface, as well as canines and molars. The main characteristic is that the hard structures of the teeth are affected by microstructural loss of mineralized tissue in areas where high levels of stress are concentrated.

Bhundia et al. [83] further emphasize in a recent literature review that there is also no unanimous consensus among clinicians on whether occlusal loading can generate sufficient tensile stress to be the sole and only etiological factor responsible for the loss of hard dental tissue at the enamel-cement junction, indicating a multifactorial etiology for the lesions referred as abfraction [83]. These considerations have been repeatedly addressed in the past decades; in fact, in 2009, Bartlett indicated the presence of little clinical evidence for abfraction, apart from laboratory studies, indicating the existence of abfraction only as a hypothetical component of wear cervical [83].

According to the abfraction theory, the loss of tooth enamel structure is due to bending forces, which are concentrated in the region of the cementoenamel junction (CEJ).

The detachment of some parts of the enamel induced by the resulting forces at the level of the CEJ causes the destruction of the enamel layer [84].

The abfraction appears to be generated by endogenous physical–mechanical causes, such as an incorrect occlusion with precontacts, clenching, or bruxism [85, 86].

On the other hand, abrasion appears to be conditioned by exogenous physical and mechanical stimuli, such as incorrect brushing and the use of hard bristles, which induce either excessive brushing force or a combination of the previous ones [87].

The dental elements that are subjected to bending forces, produced during occlusion, are subjected to tension and compression action on dental element opposite sites.

In this case, the tooth must be able to manage the forces balance and to discharge them until they produce loss and integrity of the enamel, with the V-shaped lesions on the side where the compressive forces tension and C-forces converge [88], leading in this way the enamel to produce possible microcracks, which predispose the surfaces to abrasion [89].

The microfractures that give rise to cervical lesions in abfraction are believed to be generated by nonaxial occlusal forces, which result in compression stress on one side and tensile stress on the opposite side. These cyclic solicitations lead to the formation of fissures and ruptures that separate the enamel prisms and increase susceptibility to erosion in the cervical areas. The applied forces, causing the rupture of hydroxyapatite crystals, allow small molecules and water to penetrate between the prisms, hindering the restoration of interprismatic bonds upon the cessation of force application. These mechanisms render hydroxyapatite crystals more susceptible to erosive and abrasive phenomena, leading to the described lesions attributed to abfraction. In fact, the ultimate effect of this process is the loss of mineralized tissue in the tooth (enamel and subsequently dentin) [90].

Patients who carry out particular oral hygiene maneuvers can favor the appearance of abfraction also because the most frequent localizations occur on the vestibular face of the upper molars and premolars, both areas in which patients use the greatest brushing forces [91, 92].

Furthermore, abfractions usually also involve adjacent teeth, while stress-induced cervical lesions only affect elements with a longitudinal axis oriented to favor the development of eccentric occlusal loads during functional and parafunctional mandible movements [93].

Many literature evidence in support of the abfraction theory comes from laboratory studies, according to evidence-based dentistry [94]. In fact, a systematic literature review conducted by Duangthip et al. [16] investigating the potential association between occlusal stress and the formation of NCCLs, identifying a total of 38 laboratory studies, of which 33 did not exclude a possible association, indicating occlusal stress as a potential mechanism for NCCLs formation [16]. Specifically, a study by Soares et al. [95], considering 3D finite element analysis, demonstrated in vitro that, during deep lesions, non-axial occlusal loading resulted in a higher concentration of stress and deformation [95]. These findings were further confirmed in another laboratory study conducted by Jakupovic et al. [96], which found that occlusal loading, in addition to the contact region, leads to significant stress in the tooth cervical part. In more detail, the subsurface layer of cervical enamel was found to be more affected by stress, with applied forces reaching values five times higher compared to the superficial enamel layers [96].

## 4. Conclusions

The processes underlying the erosive and abrasive phenomena are widely discussed and clarified, while those related to the presumed phenomena described as abfraction are not entirely and fully clinically demonstrated, so, is necessary further clarification, which must be investigated with longitudinal randomized studies with control groups.

## Figures and Tables

**Table 1:** Gradation of the vestibular surface.

Grade	Tissue status
0	Absence of erosion, the tooth appears free from imperfections

1	Loss of superficial enamel. The lesion edges appear wavy without dentin involvement

2	Dentinal involvement for less than half of the tooth surface

3	Dentin involvement for more than half of the tooth surface

**Table 2:** Gradation of the oral and occlusal surfaces.

Grade	Tissue status
0	We do not notice any erosive lesions; The tooth surface is smooth and shiny

1	Presence of a small erosion; Loss of superficial enamel; The dentin does not appear to be involved

2	Severe erosions, more marked signs of the first degree, and dentin involvement
